# Influence of the thickness of an antiferromagnetic IrMn layer on the static and dynamic magnetization of weakly coupled CoFeB/IrMn/CoFeB trilayers

**DOI:** 10.3762/bjnano.9.206

**Published:** 2018-08-20

**Authors:** Deepika Jhajhria, Dinesh K Pandya, Sujeet Chaudhary

**Affiliations:** 1 Thin Film Laboratory, Department of Physics, Indian Institute of Technology Delhi, New Delhi 110016, India

**Keywords:** ferromagnetic resonance, interlayer exchange coupling, magnetic damping, magnetic thin films, spin pumping

## Abstract

The static and dynamic magnetization response of the CoFeB/IrMn/CoFeB trilayer system with varying thickness of the antiferromagnetic (AF) IrMn layer is investigated using magnetization hysteresis (*M*–*H*) and ferromagnetic resonance (FMR) measurements. The study shows that the two CoFeB layers are coupled via a long-range dynamic exchange effect through the IrMn layer up to a thickness of 6 nm. It is found that with the increase in IrMn layer thickness a nearly linear enhancement of the effective magnetic damping constant occurs, which is associated with the simultaneous influence of spin pumping and interlayer exchange coupling effects. An extrinsic contribution to the linewidth originating from the two-magnon scattering is also discussed. The AF-induced interfacial damping parameter is derived by studying the evolution of damping with inverse CoFeB thickness. The static magnetic measurements also reveal the interlayer exchange coupling across the IrMn layer both at room temperature and low temperature. The asymmetric hysteresis loop and training effect observed at low temperature is related to the presence of a metastable AF domain state. We show that both the static and dynamic magnetic properties of trilayer films can be adjusted over a wide range by changing the thickness of the IrMn spacer layer.

## Introduction

Traditionally, antiferromagnets (AF) are known to play only a static role by pinning adjacent ferromagnetic (FM) layers via exchange bias in spin-valve devices [1]. Recently, AF-based spintronics is gaining momentum because of the unique properties such as zero net magnetization, no stray fields, low magnetic susceptibility, large spin–orbit coupling, ultrafast dynamics and large magneto-transport effects [2–6]. Several of the effects such as tunnel anisotropic magnetoresistance [7], inverse spin Hall effect [8] and spin Seebeck effect [9–10] have already been reported in AF materials.

Transfer of spin angular momentum presents one of the promising ways to control the magnetic properties of FM thin films [11]. However, little is known about spin transport in AF materials. There are theoretical studies that suggest the possibility of manipulating the AF moment by spin transfer torque [12–14]. Only recently, FM/AF (NiO, IrMn)/heavy metal heterostructures have been extensively studied to demonstrate the efficient spin current transfer across the AF layer mediated by AF-magnon propagation [15–21]. Besides spin transport, it is important to understand and tune the magnetic relaxation in multilayers from both fundamental physics and technological viewpoints. There are many applications that require low damping of gyromagnetic precession, e.g., in lowering the spin torque critical current switching density [22–23]. For various other applications a fast relaxation is favored, e.g., in magnetoresistive heads [24–25]. The FM/AF exchange coupling is one of the approaches to tune the magnetization relaxation by enhancing the field linewidth Δ*H* and displacing the resonance field *H*_r_ [26–29].

Spin transport and relaxation studies in FM/AF/FM trilayers are intriguing due to the presence of two interfaces shared between FM and AF. Also, little is known about how effective magnetic damping evolves when there is a weak interlayer exchange coupling between two FM layers through an AF layer. Motivated by this, we chose a rather uncommon CoFeB(10 nm)/IrMn(*t*_IrMn_)/CoFeB(10 nm) trilayer system to investigate the interlayer exchange coupling, spin transport, magnetic damping and magnetization reversal by carrying out ferromagnetic resonance (FMR) and magnetization hysteresis (*M*–*H*) measurements.

We find compelling evidence that the two CoFeB layers are dynamically exchange-coupled through the IrMn spacer layer up to a thickness *t*_IrMn_ = 6 nm. The dynamic exchange coupling is discussed in terms of interaction between two CoFeB layers mediated by the IrMn layer via conduction electrons and also by the propagation of magnetic excitations. FMR measurements are used to quantify magnetic damping, which is directly associated with the interaction between AF moments and spin current. The magnitude of the effective Gilbert damping constant (α_eff_) of trilayers shows rapid enhancement with the increase in *t*_IrMn_. We explain this increase in damping as a combined effect of spin pumping and interlayer exchange coupling. The spin wave relaxation is explained by taking into consideration the intrinsic as well as extrinsic contributions to the linewidth. In addition, the AF-induced interfacial damping parameter is also calculated. At low temperatures, the asymmetric hysteresis loop and training effect indicates the presence of a dynamic AF spin structure instead of a static structure.

## Experimental

FM/AF/FM trilayers of Co_20_Fe_60_B_20_ (10 nm)/Ir_19_Mn_81_ (*t*_IrMn_)/Co_20_Fe_60_B_20_ (10 nm) were deposited at room temperature using pulsed-DC magnetron sputtering on naturally oxidized Si wafers with Ta (5 nm) seed and cap layers. The base pressure and working pressure of deposition were 2·10^−7^ and 4·10^−3^ Torr, respectively. The thickness *t*_IrMn_ was systematically varied in 1 nm steps from 0 to 7 nm. The IrMn spacer layer was deposited at a lower growth rate (i.e., 0.17 Å/s compared to 1.3 Å/s for CoFeB) for better uniformity and lower interfacial roughness. In another sample series, the *t*_IrMn_ was kept constant at 2 nm while the thickness of the top and bottom FM layers *t*_CoFeB_ was varied (5, 10, 15, 20 and 25 nm). No cooling of the samples in an external magnetic field through the Néel temperature (*T*_N_) was performed.

For accurately estimating the thicknesses of individual layers and interfacial roughness, X-ray reflectivity (XRR) measurements were performed using a PANalytical X-Pert PRO diffractometer. The XRR spectra were simulated using the WinGixa software based on genetic algorithms (version 1.102). The static magnetic properties of trilayers were characterized using the physical property measurement system (PPMS) (Quantum Design Inc, EverCool II) with vibrating sample magnetometer (VSM) option. The magnetization dynamics of the films was studied using a broadband (5–13 GHz) ferromagnetic resonance setup employing a vector network analyzer (VNA) and co-planar waveguide (CPW) transmission line. Since the output microwave power of the VNA was set to 0 dBm, the amplitude of magnetization precession is small, which results in a nearly linear FMR response and thus the complicated non-linear magnetization dynamics is less likely in the films. The samples were mounted with the deposited film side directly facing down on to the CPW. The FMR spectra were recorded by sweeping the in-plane external dc magnetic field through resonance at a constant microwave frequency (*f*). Here, the external field was modulated using a pair of Helmholtz coils, which provide a small ac field (211.5 Hz) of 1.3 Oe, and in combination with lock-in detection, the field derivative of FMR absorption is obtained. For each frequency, the values of *H*_r_ and Δ*H* and were extracted from the fitting of the FMR spectra with the derivative of the Lorentzian function.

## Results and Discussion

**Figure 1:**
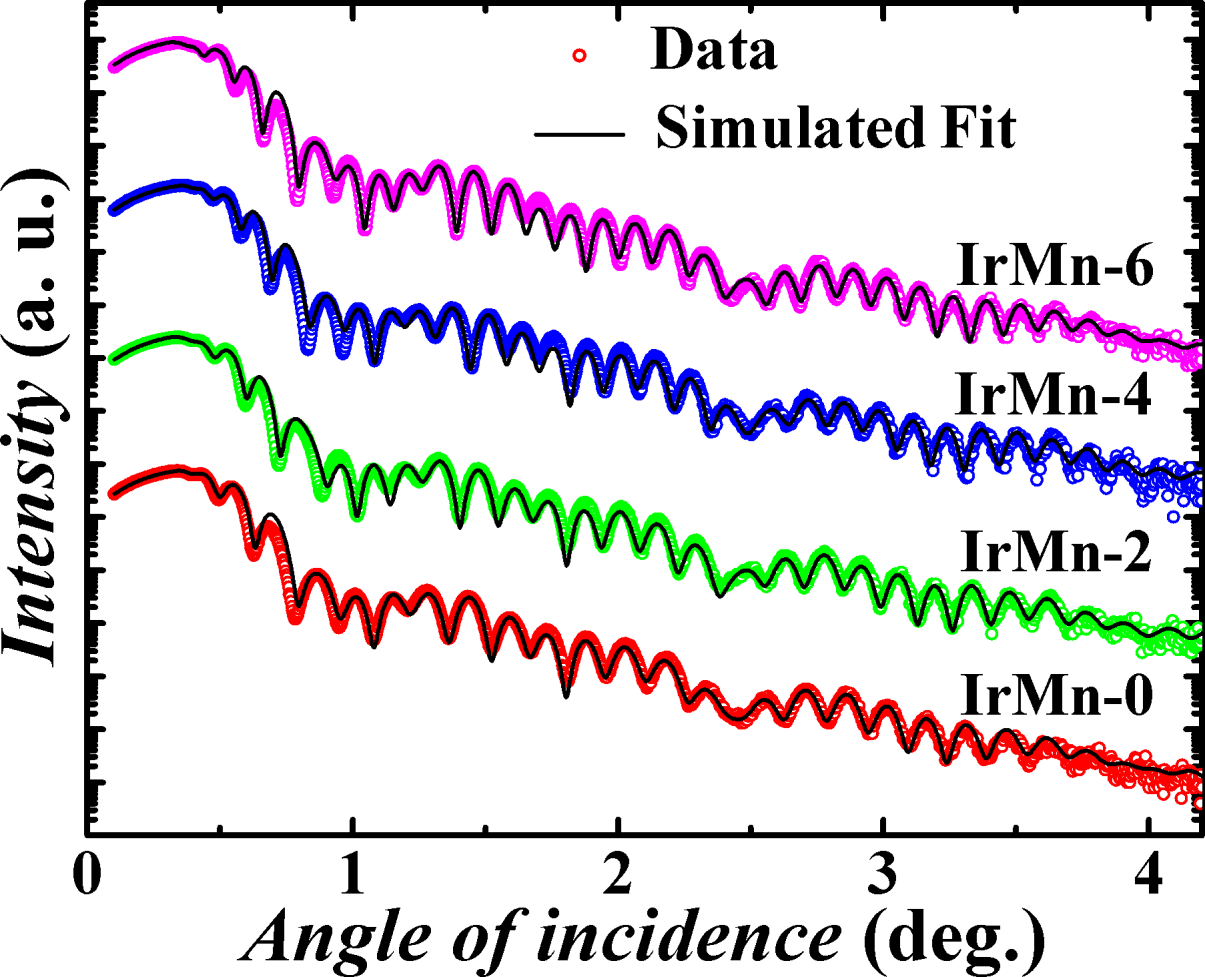
Specular XRR spectra of CoFeB/IrMn/CoFeB trilayers with *t*_IrMn_ = 0, 2, 4 and 6 nm.

The XRR spectra and their simulated fits for trilayered films with *t*_IrMn_ = 0, 2, 4 and 6 nm are shown in Figure 1. For all the samples, distinct Kiessig fringes appeared over a wide range of the incidence angle (ca. 4°). This indicates the presence of sharp interfaces and excellent sample quality. The summary of the fitting results is presented in Table 1. The interfacial width between top CoFeB and underlying IrMn layer depends on the thickness of the IrMn layer. It is evident that the IrMn interfacial roughness is slightly higher for *t*_IrMn_ ≥ 5 nm. Below 5 nm, the interface is quite smooth with roughness values of about 0.5 nm. This increase in interfacial roughness might be arising due to the increase in the crystallinity and hence the increase in mean grain size of IrMn at higher thickness. The average interfacial roughness of the top and bottom CoFeB layers was ca. 0.6 nm, and that of the Ta seed and cap layers is below 0.5 nm for all the samples.

**Table 1:** Fitting parameters obtained from the simulation of specular XRR spectra recorded on Si/Ta (5 nm)/CoFeB (10 nm)/IrMn (1–7 nm)/CoFeB (10 nm)/Ta (5 nm) multilayer structures.

sample	nominal *t*_IrMn_ (nm)	simulated *t*_IrMn_ (nm) (±0.01 nm)	top CoFeB/IrMn interface width (±0.05 nm)

IrMn-1	1	0.50	0.38
IrMn-2	2	2.15	0.39
IrMn-3	3	3.25	0.56
IrMn-4	4	4.75	0.40
IrMn-5	5	5.68	0.87
IrMn-6	6	6.63	1.01
IrMn-7	7	7.57	1.02

**Figure 2:**
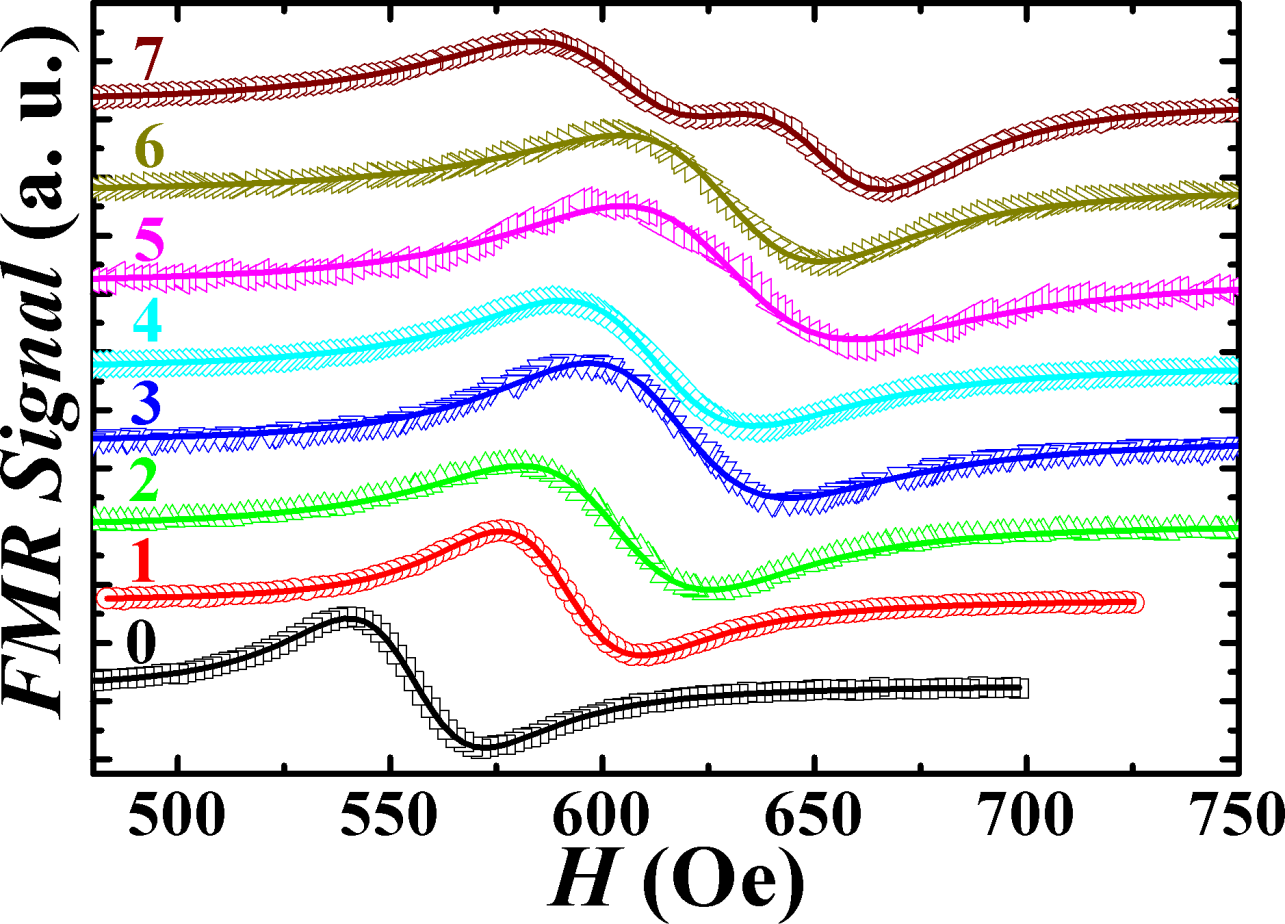
The field-swept FMR spectra for the sample series recorded at 9 GHz (numbers represent IrMn layer thickness in nanometers). Open symbols represent the experimental data, and the solid lines are fits to a Lorentzian function.

The FMR measurements allow us to investigate the interaction between spin current and AF magnetic moment. Figure 2 shows characteristic FMR spectra of the trilayer sample series recorded at 9 GHz. For *t*_IrMn_ ≤ 6 nm, only one spectral peak is observed. It indicates the presence of a long-range dynamic exchange coupling between the two CoFeB layers across the IrMn spacer layer, which prevents them from precessing independently such that each of the FM layer drags the magnetization of another FM layer. The FMR peak is well fitted using the Landau–Lifshitz (LL) equation for single mode and is attributed to the acoustic FMR mode whereby the two dynamically exchange-coupled CoFeB layers undergo in-phase precession across the IrMn spacer layer. It is to be noted that it is highly unlikely that the observed spectral peak corresponds to two independent uncoupled degenerate modes with equal linewidth since the seed and cap layer configuration are different for the two involved CoFeB layers (i.e., Ta/CoFeB/IrMn and IrMn/CoFeB/Ta). This would result in unequal linewidths and a deviation from the LL equation fit. The interlayer exchange coupling could not be quantified in our case since the optical mode (anti-phase precession) intensity is too small to be detected. This is because the two FM layers are magnetically identical giving rise to the presence of a dominant acoustic mode and a weak optical mode [30–31]. For *t*_IrMn_ = 7 nm, the decoupling occurs as indicated by the presence of two independent FMR modes with slightly different values of *H*_r_ and Δ*H* corresponding to each CoFeB layer.

The dynamic exchange interaction occurring between CoFeB layers across the IrMn spacer layer is interpreted in terms of transfer of non-equilibrium spin current through the IrMn layer and could be explained by two possible mechanisms. One is that the spin current generated from the precessing CoFeB can propagate across the AF layer with the help of conduction electrons in the IrMn layer. However, since the spin diffusion length *l*_s_ of conduction electrons in the IrMn layer is very small (ca. 1.4 nm) [32–33], this mechanism of interlayer coupling is not expected beyond 2 nm thickness of the IrMn layer.

According to the theoretical calculations [34], the intrinsic magnetic ordering of the AF materials can itself sustain propagating spin excitations thereby potentially allowing the transport of spin angular momentum. These spin excitations exist in the form of AF magnons (excitation states of ordered AF spins, when *T*_N_ is above the measurement temperature) and AF spin fluctuations (excitation states of dynamically correlated AF spins, when *T*_N_ is below the measurement temperature). It is also equally important to understand the mutual interconversion of spin current and AF magnons at the FM/AF interface since the energy of a uniform precession magnon (wave vector *k* = 0) is too small to excite any AF magnons (frequency in the terahertz range). According to theoretical studies, the coupling between FM magnons and AF magnons across the interface takes place via at least four-magnon interaction as the two-magnon interaction is prohibited [35–36]. Therefore, we argue that there can be another alternative mechanism for the transfer of spin angular momentum in the IrMn layer based on the propagation of AF spin excitations. This can conceptually explain the long-range dynamic exchange coupling observed here for CoFeB/IrMn/CoFeB up to *t*_IrMn_ *=* 6 nm. It is noteworthy that *T*_N_ of IrMn falls below room temperature for *t*_IrMn_ ≤ 2.7 nm, indicating the prominent role of AF spin fluctuation in spin transport for this thickness range [37–38]. A similar dynamic spin transport through metallic IrMn layers of up to 3 nm of thickness via AF spin excitations has also been previously reported in Pt/IrMn/YIG heterostructures using spin-torque FMR [17]. Also, a highly efficient spin current transfer from Y_3_Fe_5_O_12_ (YIG) into NiO and subsequent spin propagation in NiO with spin decay lengths (healing length) of up to 10 nm has been reported by Wang and co-workers [15]. Our results do suggest that the IrMn moments are interacting with the spin current, while no direct information about spin transport is presented. Therefore, there is still a critical need for further theoretical and experimental investigations for the complete understanding of spin transport within the AF layer and across FM/AF interface.

**Figure 3:**
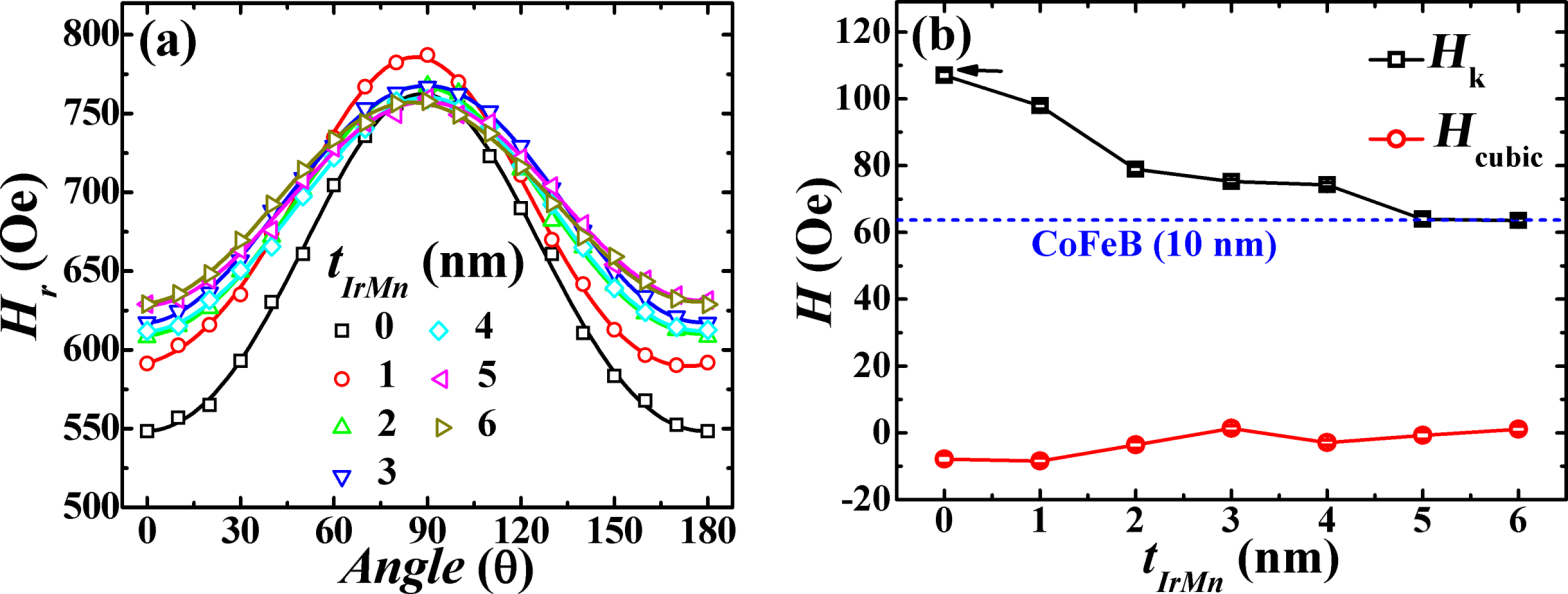
(a) Resonance field *H*_r_ as a function of the angle θ at 9 GHz. Open symbols and solid lines represent the experimental data and the fit to Equation 1, respectively. (b) *H_k_* and *H*_cubic_ as functions of *t*_IrMn_. The horizontal dashed line corresponds to the *H_k_* value measured for a 10 nm thick single CoFeB layer.

In-plane angle-dependent FMR measurements of the exchange coupled trilayers were performed at 9 GHz. In Figure 3a, we have plotted the obtained resonance field *H*_r_ as a function of the angle θ. The two-fold symmetry for all samples clearly indicates the existence of the uniaxial magnetic anisotropy (UMA). This UMA results from the breakdown of the azimuthal symmetry in the deposited films, which is likely to be caused by the anisotropic stress in the films generated due to the oblique deposition geometry [39]. Since CoFeB is highly sensitive to this induced anisotropic stress (owing to their large positive saturation magnetostriction coefficient λ_s_ [40]), the deposition geometry effectively results in the observed UMA [41]. The UMA in films is found to be directly proportional to the thickness of the CoFeB layers (up to 50 nm, data not shown), demonstrating that the UMA is a volume anisotropy rather than an interfacial anisotropy.

From the fitting of the angular data, the individual values of uniaxial and cubic anisotropy field were calculated with the following equation [42]:

[1]
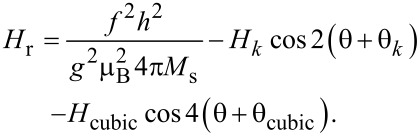


Here, *h* is Planck’s constant, 4π*M*_s_ is saturation magnetization, *g* is the Landé g-factor, *H_k_* and *H*_cubic_ are the uniaxial and cubic anisotropy fields, respectively, and θ*_k_* and θ_cubic_ are the angles between, respectively, uniaxial and cubic anisotropy easy axes and the observed easy axis (EA, θ = 0). The values of *H_k_*, *H*_cubic_, θ*_k_*, θ_cubic_ and g^2^4π*M*_s_ were obtained as fitting parameters. From Figure 3b, it can be seen that *H_k_* is far more dominant than *H*_cubic_ in all samples. For *t*_IrMn_ = 0 nm (which, in fact, is a single layer CoFeB of thickness 20 nm), a *H_k_* value of 106 Oe (marked by a horizontal arrow in Figure 3b) was found. On introducing the IrMn spacer layer, the *H_k_* values initially show a rapid decrease, followed by a gradual decrease at higher *t*_IrMn_, such that *H_k_* approaches the value observed for a 10 nm thick single CoFeB layer in the limit of an infinite IrMn spacer layer thickness. The presence of long-range dynamic exchange coupling can support this observed trend of *H_k_* in trilayers as it is natural to suppose weaker interlayer exchange coupling in FM/AF/FM systems at larger thicknesses of the AF layer.

**Figure 4:**
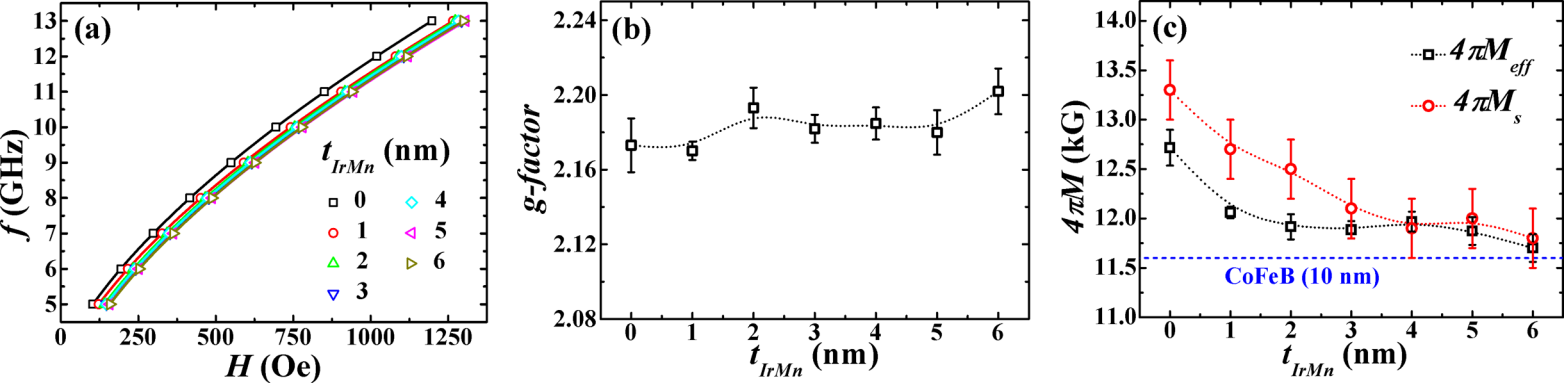
(a) *H*_r_ as a function of *f* for the entire sample series*.* Open symbols and solid lines represent the experimental data and the fit to Equation 2, respectively. (b) Variation of *g* with *t*_IrMn_. (c) Dependence of 4π*M*_eff_ and 4π*M*_s_ on *t*_IrMn_. The lower horizontal dashed line corresponds to the 4π*M*_eff_ value measured for a 10 nm thick single CoFeB layer. The dotted lines are guides to the eye.

Subsequently, the acoustic FMR spectra of the films were recorded along the EA at different *f* in the range of 5–13 GHz. The obtained plots of *H*_r_ as a function of *f* (Figure 4a) were fitted using the Kittel equation [43]:

[2]
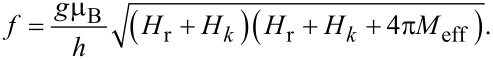


Here, 4π*M*_eff_ is the effective magnetization. In order to determine *g* values more accurately, we used the previously determined values of *H_k_* (from the angular FMR measurements) in Equation 2 to obtain g and 4π*M*_eff_ values as fitting parameters [41,44]. The resulting *g* values in the films (Figure 4b) are comparable to the already reported *g*-factor values for CoFeB thin films, and the higher values are associated with larger orbital contributions due to the presence of the high anisotropy field [44–45]. The *g* values of the trilayer films do not vary much with *t*_IrMn_ and their influence on the properties related to spin–orbit coupling (SOC), such as magnetic anisotropy and damping is not expected to be significant.

The effective magnetization is related to the interfacial properties by the relation 4π*M*_eff_ = 4π*M*_s_ − (2*K*_s_/*M*_s_)*t*_FM_^−1^, where *K*_s_ is the surface anisotropy constant, 4π*M*_s_ is the saturation magnetization and *t*_FM_ is the FM layer thickness. For the CoFeB thickness used in the present study (10 nm), the contribution of the interface is negligible and thus 4π*M*_eff_ essentially follows the trend of 4π*M*_s_ (extracted from VSM measurements) as shown in Figure 4c. The 4π*M*_eff_ values fall off rapidly for *t*_IrMn_ ≤ 2 nm and remains almost unchanged afterwards. At higher *t*_IrMn_, the value of 4π*M*_eff_ approaches that of a 10 nm thick single CoFeB layer. We infer that the decreased value of 4π*M*_eff_ for higher *t*_IrMn_ could result from the weakening of interlayer exchange coupling in the CoFeB/IrMn/CoFeB system with each of the CoFeB layers tending to behave as an isolated 10 nm thick single CoFeB layer [46]. It is remarkable that both *H_k_* and 4π*M*_eff_ exhibit a comparable dependence on *t*_IrMn_ (compare Figure 3b and Figure 4c) further suggesting that the observed interdependence of *H_k_* and 4π*M*_eff_ has a common origin in the *t*_IrMn_-dependent interlayer exchange coupling.

**Figure 5:**
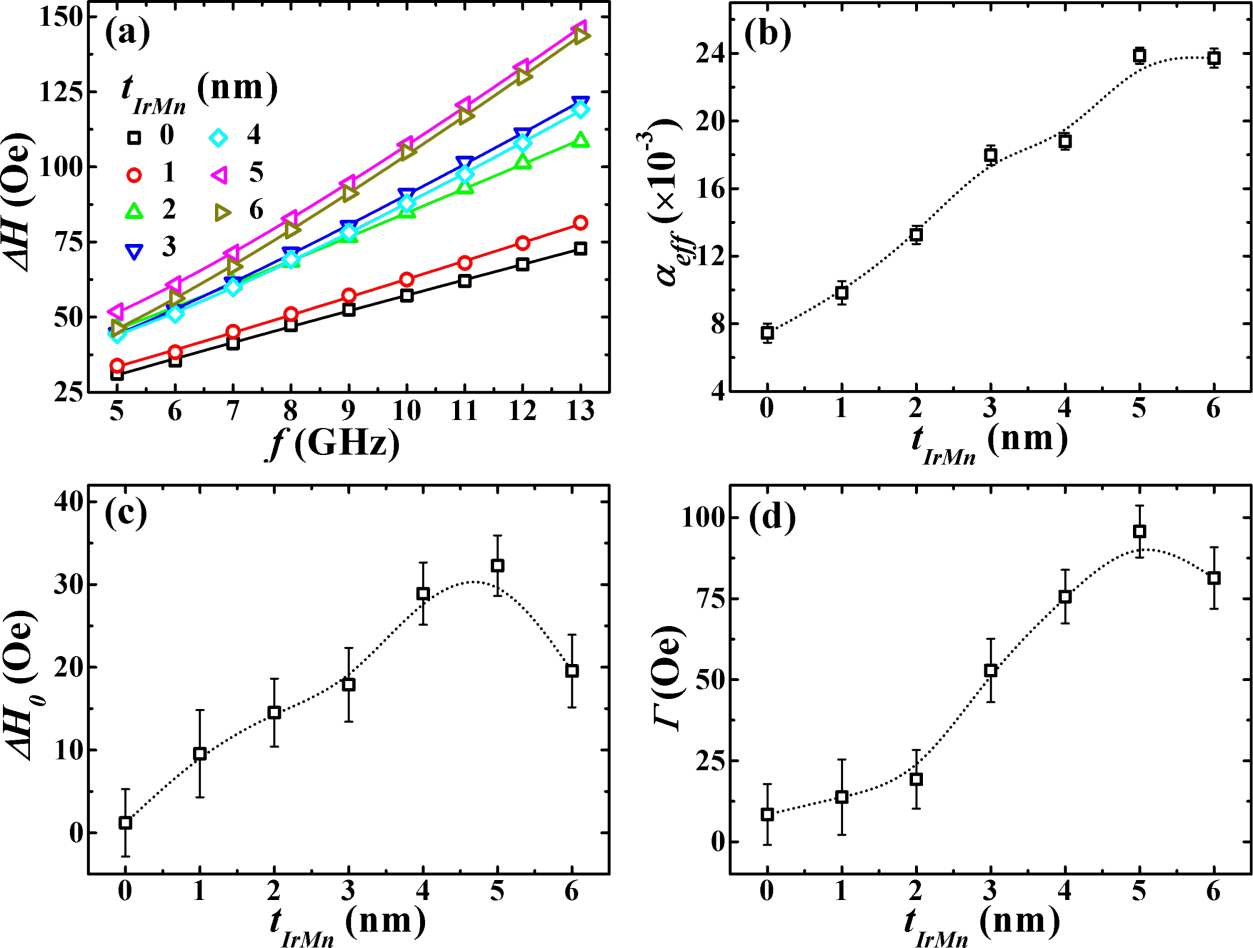
(a) Δ*H* as a function of the frequency with open symbols representing the experimental data and solid lines are fit to Equation 3 and Equation 4. (b) Dependence of α_eff_ on *t*_IrMn_. (c) Variation of Δ*H*_0_ as a function of *t*_IrMn_. (d) Dependence of TMS strength (Γ) on *t*_IrMn_. The dotted lines are guides to the eye.

To gain insight into the various magnetic relaxation mechanisms in trilayers, the frequency dependence of Δ*H* is plotted in Figure 5a. There is an almost linear increase of the Δ*H* values with frequency, which indicates the predominance of damping through magnon–electron scattering [47]. However, for exchange-coupled trilayers an additional extrinsic relaxation channel due to two-magnon scattering (TMS) must be taken into consideration for accounting the slight nonlinearity observed in the Δ*H*(*f*) plots. In TMS, the precession of the uniform mode of magnetization (*k* = 0) is scattered into degenerate state non-uniform spin wave modes (k ≠ 0) mediated by sample inhomogeneities or defects [48–49].

Therefore, the total contribution to the observed linewidth must include both intrinsic and extrinsic relaxation terms:

[3]
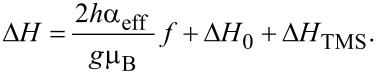


Here, the first term represents the linewidth broadening due to Gilbert damping with α_eff_ as the dimensionless effective Gilbert damping constant. The second term Δ*H*_0_ is the inhomogeneous linewidth broadening (zero-frequency intercept), and the last term Δ*H*_TMS_ is the linewidth contribution from TMS which has the following frequency dependence [50]:

[4]
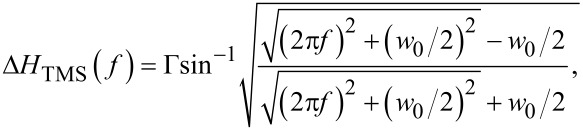


where


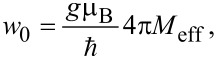


and the prefactor Γ represents the strength of the TMS. The Δ*H*(*f*) data was fitted with Equation 3 and Equation 4, and the values of α_eff_, Δ*H*_0_, and prefactor Γ were obtained as fitting parameters.

Figure 5b clearly shows the strong dependence of α_eff_ on *t*_IrMn_. The damping is increased more than threefold (from 0.00745 ± 0.0005 for *t*_IrMn_ = 0 nm to 0.02372 ± 0.0005 for *t*_IrMn_ = 6 nm). It may be pointed out that the observed damping in coupled trilayer samples is far larger compared to that found in a 10 nm thick single CoFeB layer (α_eff_ = 0.0094). We interpret this damping enhancement as a result of several mechanisms. It is in part due to the transfer of spin angular momentum from the precessing CoFeB layer into the adjacent IrMn layer (which has strong SOC and a non-collinear magnetic structure) by spin pumping [51–52]. This is a very likely mechanism since *t*_IrMn_ is thicker than *l*_s_. Hence, the IrMn layer can effectively absorb the spin current. Also, it is interesting to note that the damping in weakly coupled trilayers is way higher than that measured in trilayers with uncoupled precession, i.e., *α*_eff_ = 0.011 and 0.010 for the two uncoupled CoFeB layers. Therefore, the enhanced damping observed in coupled trilayers cannot be associated with spin pumping alone, and it is equally important to consider the linewidth broadening based on interlayer exchange coupling mediated by the AF spin structure. The weak interlayer exchange coupling due to the large separation of the CoFeB layers causes the magnetization in each layer to precess almost independently. Each CoFeB layer resonance precession drags the magnetization of the other layer and the obtained FMR spectra are the superposition of resonance peaks originating from the two CoFeB layers. This resonance mode hybridization does not result here in two resonance modes with different linewidth but could itself be a source of damping enhancement especially for higher *t*_IrMn_ [53]. Also, in trilayers, the spin angular momentum can experience an additional damping dissipation at the FM/AF interface resulting from the direct exchange coupling between FM and AF layers [29], which in our case is minimal as evidenced by the absence of exchange bias at room temperature (discussed below). So, the enhanced damping results from the simultaneous influence of spin pumping and weak interlayer exchange-coupling effects, and it is difficult to separate their individual contributions from the current experiment. We can safely say that the Gilbert damping in interlayer exchange coupled trilayers can be modified over large range with varying *t*_IrMn_, and it is also higher compared to that of uncoupled trilayers and single layers.

The values of Δ*H*_0_ of the trilayer system also increase rapidly with increasing *t*_IrMn_ as shown in Figure 5c. The almost linear rise of Δ*H*_0_ suggests an increased spatial dispersion in the magnitude and direction of both magnetization and magnetic anisotropy. It could be associated with the magnetic disorder created due to the large interfacial roughness of IrMn at higher *t*_IrMn_, which is also supported well by the XRR fitting results. On the other hand, the extrinsic contribution to the linewidth through TMS (quantified with Γ) is shown as a function of *t*_IrMn_ in Figure 5d. For small *t*_IrMn_, the prefactor Γ showed a very gradual increase, with values as low as 20 Oe for *t*_IrMn_ ≤ 2 nm, suggesting relaxation via mainly intrinsic damping. At higher *t*_IrMn_ , the Γ showed a profound increase due to the fluctuation of local exchange coupling arising from the increased IrMn interfacial roughness [27]. Therefore, it is found that the IrMn interfacial roughness plays critical role in the variation of inhomogeneous broadening and two-magnon scattering and thus influences the overall damping.

**Figure 6:**
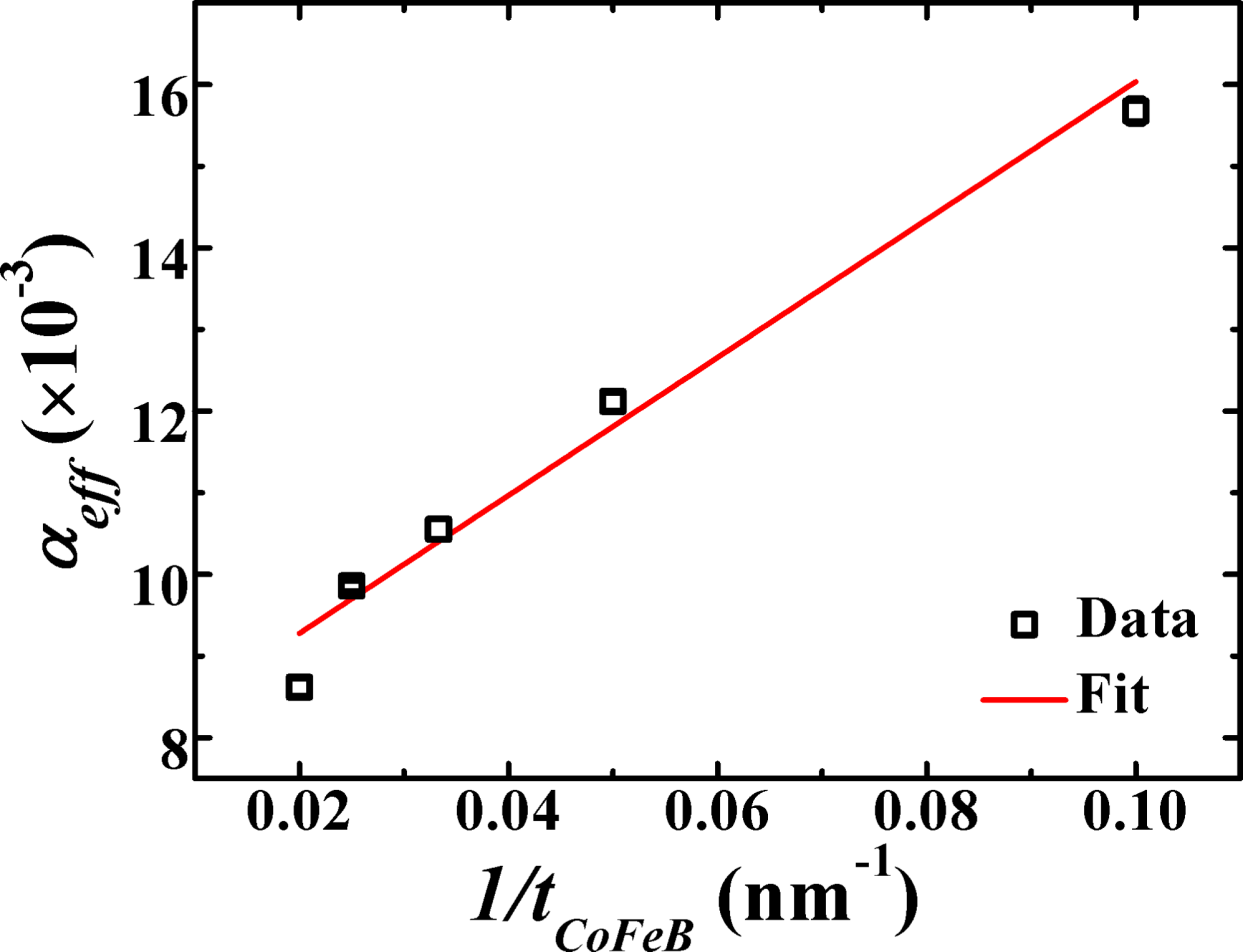
α_eff_ as a function of the inverse FM layer thickness 1/*t*_CoFeB_ for different CoFeB/IrMn (2 nm)/CoFeB structures.

In the following, the FMR results of trilayers deposited with a constant IrMn layer thickness (2 nm) and variable CoFeB thickness *t*_CoFeB_ will be discussed. The linear dependence of α_eff_ on 1/*t*_CoFeB_ (Figure 6) demonstrates a significant contribution of the interfacial Gilbert damping. This clearly suggests that spin pumping (an interfacial effect) is the dominant mechanism of enhanced damping for a given IrMn thickness. The α_eff_ is described by the following equation [54]:

[5]
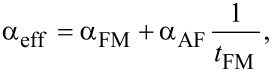


where α_FM_ is the intrinsic damping contribution of the FM layer, and α_AF_ the interfacial damping coefficient of the AF layer. We extracted values of α_AF_ ≈ 0.08·10^−9^ m^−1^ and α_FM_ = 0.00749 ± 0.0003 from the fit.

**Figure 7:**
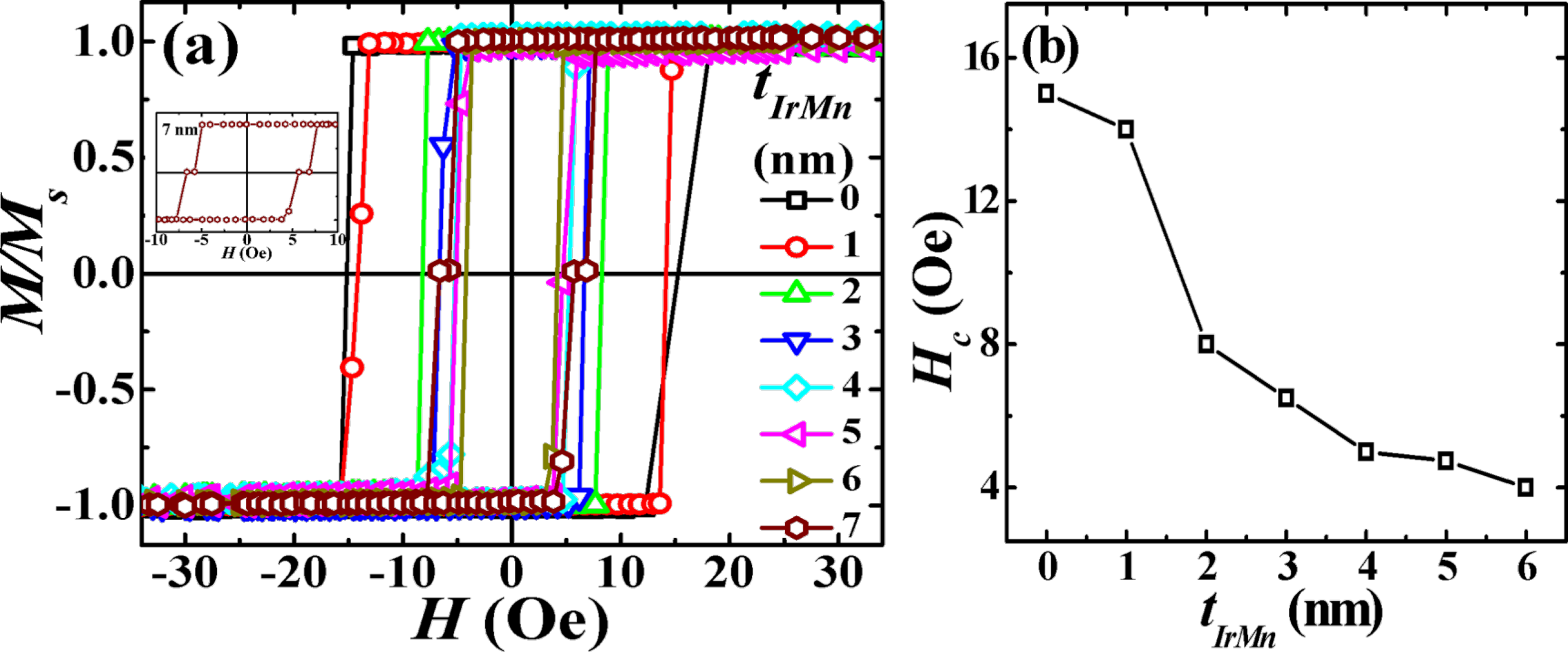
(a) The easy-axis *M*–*H* loops of trilayers with varying *t*_IrMn_ recorded at room temperature (inset showing the zoomed *M–H* loop of the trilayer with *t*_IrMn_ = 7 nm). (b) *H*_c_ as a function of *t*_IrMn_.

The static magnetic measurements were performed at room temperature as well as at lower temperature in order to confirm the long-range magnetic interlayer coupling between the CoFeB layers and also to study the AF spin structure and interfacial exchange coupling in the trilayers. The *M*–*H* loops of the trilayer samples recorded at room temperature (RT) for different *t*_IrMn_ are presented in Figure 7a. A single square-shaped loop (*M*_r_/*M*_s_ ≈ 1) is obtained for *t*_IrMn_ ≤ 6 nm, which indicates that the magnetization reversal of the two CoFeB layers is indeed magnetically coupled in the trilayer system. For *t*_IrMn_ ≥ 7 nm, the magnetization reversal of the two CoFeB layers gets decoupled as confirmed by the appearance of a kink in the *M*–*H* loop. Neither exchange bias nor loop broadening are observed at RT, which indicates a very weak interfacial exchange coupling between the FM and AF spins for all the samples. The long-range interlayer coupling and the absence of exchange bias indicate that the IrMn spins are dragged by the joint torque created by the reversal of both top and bottom CoFeB layers simultaneously, which overcomes the small IrMn anisotropy possibly as a result of its small grain size. Figure 7b shows an overall dependence of coercivity (*H*_c_) on *t*_IrMn_. There is a rapid drop in *H*_c_ values for smaller *t*_IrMn_, which is followed by a gradual decrease at higher *t*_IrMn_. This behavior can be understood from the similar trend for *H*_k_ since both coercivity and anisotropy are correlated with each other as suggested by Hoffmann [55].

**Figure 8:**
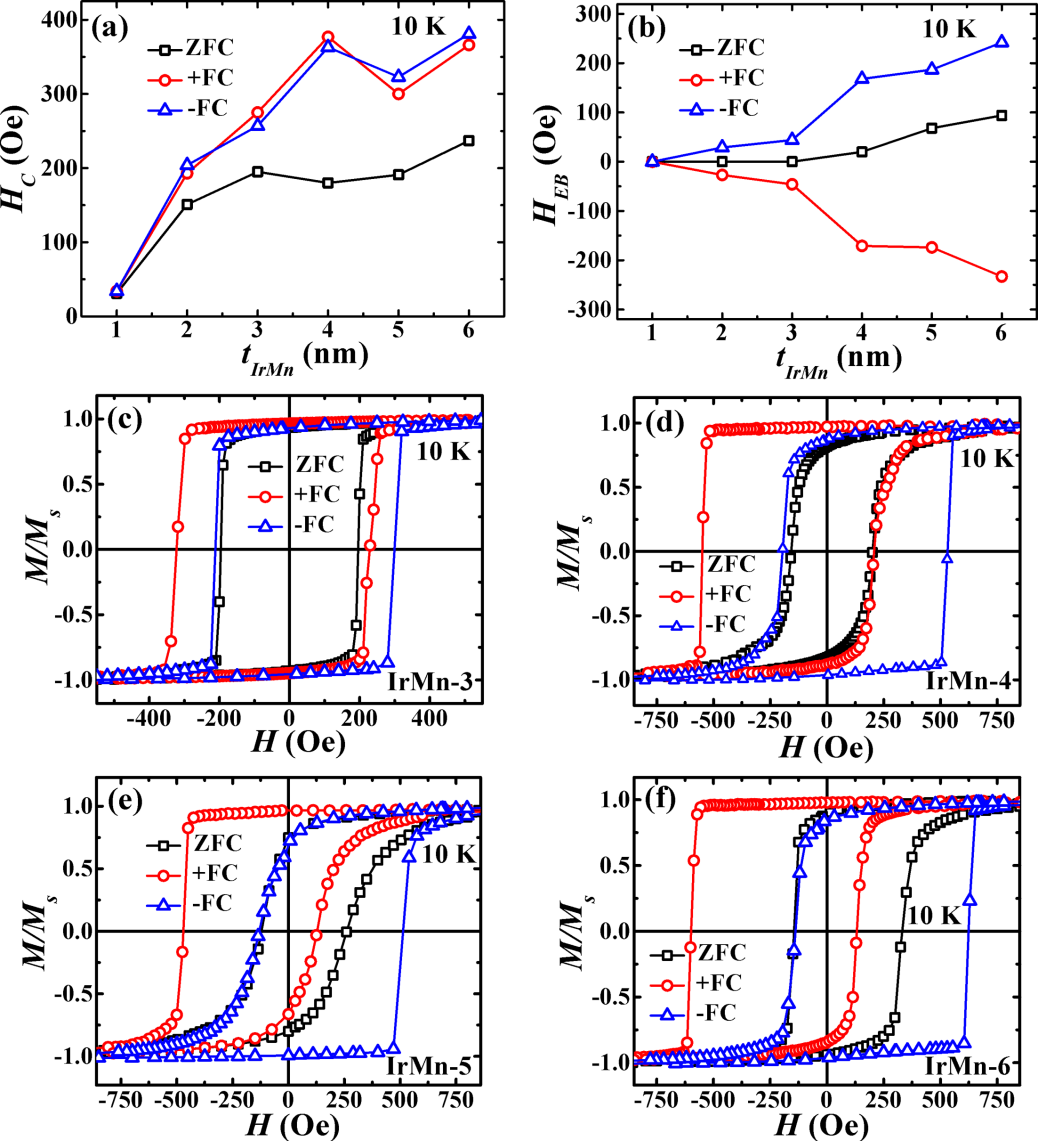
The dependence of (a) *H*_c_ and (b) *H*_EB_ on *t*_IrMn_, as extracted from the individual hysteresis loops measured along the EA at 10 K. ZFC refers to zero-field cooling and +FC and −FC refers to field cooling in +0.2 T or −0.2 T fields, respectively. The corresponding *M*–*H* loops of trilayers are also shown for (c) *t*_IrMn_ = 3 nm, (d) *t*_IrMn_ = 4 nm, (e) *t*_IrMn_ = 5 nm and (f) *t*_IrMn_ = 6 nm.

At a low temperature of 10 K reached after both zero-field cooling (ZFC) and field cooling (FC) procedures from RT, the top and bottom CoFeB layers are still magnetically coupled across the IrMn spacer layer for *t*_IrMn_ ≤ 6 nm. It may be noted that the *M*–*H* loops measured at 10 K after ZFC showed only loop broadening but no exchange bias (*H*_EB_) for *t*_IrMn_ ≤ 3 nm (see Figure 8a–c). Since IrMn is essentially paramagnetic for *t*_IrMn_ ≤ 2.7 nm, no exchange bias appears on ZFC for this thickness range. For *t*_IrMn_ ≥ 4 nm, the magnetization reversal is symmetric and more rounded, and a weak and positive exchange bias (PEB) also appears. The strength of PEB increases with further increasing *t*_IrMn_ as shown in Figure 8d–f. A similar positive exchange bias has been reported earlier in IrMn/CoFeB bilayers with *t*_IrMn_ > 10 nm at RT without any field cooling [56–57]. However, due to the lower IrMn thickness in our case, the PEB is indeed observed at low temperature after ZFC. Such PEB is thought to be a consequence of antiferromagnetic interface coupling between uncompensated Mn spins (present due to interfacial FM/AF roughness) and CoFeB spins.

**Figure 9:**
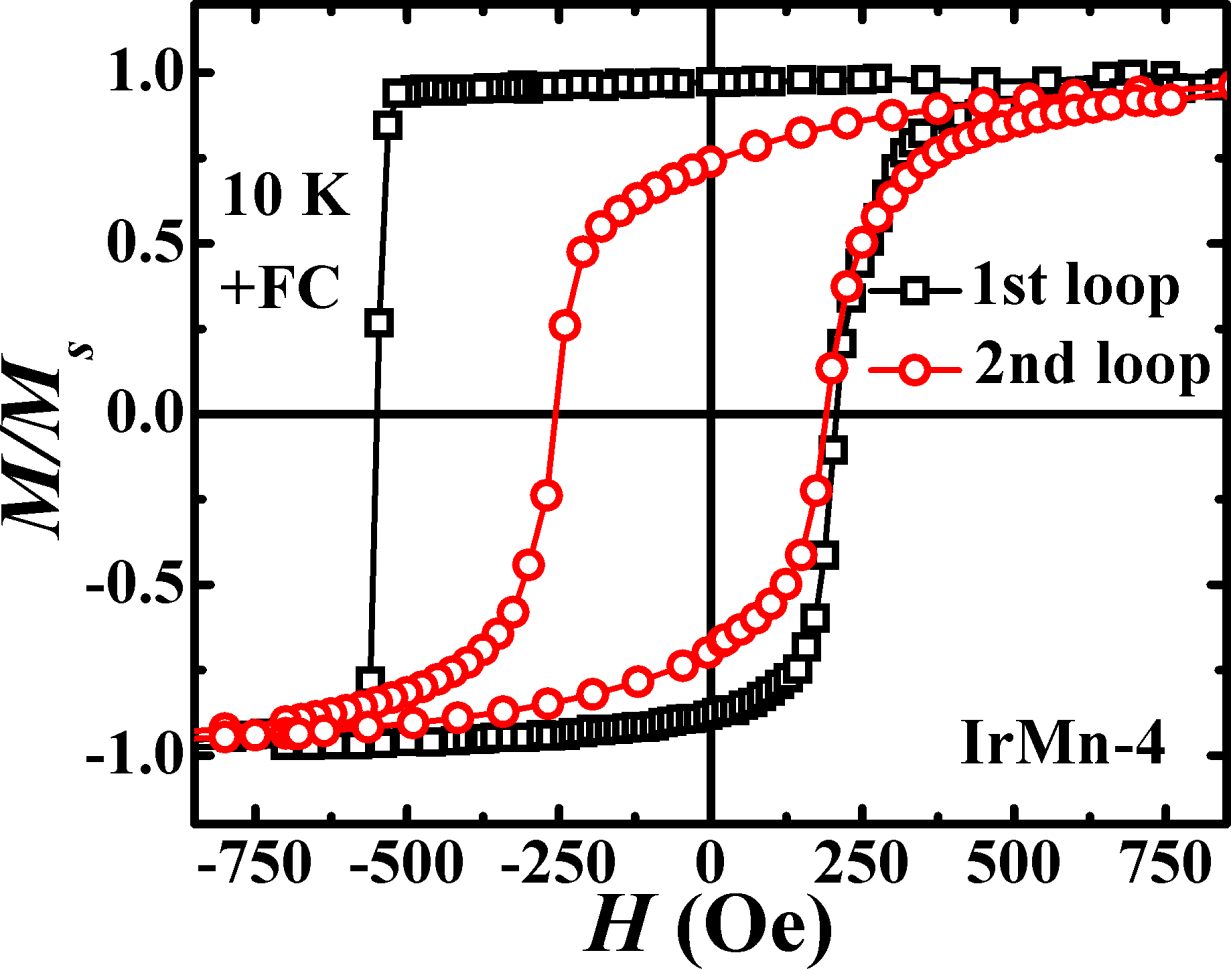
Two consecutive *M*–*H* loops for the trilayer with *t*_IrMn_ = 4 nm measured at 10 K after field cooling in +0.2 T fields.

In the case of field cooling, a large positive or negative exchange bias appears at 10 K after cooling in a negative (−0.2 T) or a positive (+0.2 T) field, respectively (Figure 8). Exchange bias and loop broadening occur at *t*_IrMn_ ≥ 2 nm, and their values continue to rise with the increase in *t*_IrMn_. Also, a profound asymmetry (c.f. the square (rounded) reversal in field decreasing (increasing) branch) in the shape of the hysteresis loops is observed for *t*_IrMn_ ≥ 4 nm. In different exchange-bias systems, such asymmetric hysteresis loops are frequently related to the training effect and its origin is explained by taking into account the irreversible changes in the AF spin configuration during magnetization reversal [58–59]. Therefore, two consecutive hysteresis loops for the trilayer with *t*_IrMn_ *=* 4 nm were measured at 10 K after field cooling in +0.2 T fields (Figure 9). We indeed found a strong training effect in the second loop, where there is large decrease in the values of coercivity and exchange bias along with a symmetrically rounded reversal at both branches of the hysteresis loop. This training results from the effect, where the initial field cooling forces the AF layer into a metastable single-domain state (characterized by a large *H*_EB_), which eventually gets destroyed during the first reversal and thereafter transforms into a stable multi-domain state in successive reversals with much smaller *H*_EB_. Thus, the observed asymmetry and training effects are incompatible with models having static AF spin structure and indicates the presence of a metastable AF domain state. Given that the reported minimum IrMn domain wall width is ca. 7.8 nm [60], which defines the length of the shortest full spiral, then in a plausible scenario the whole nominal 6 nm of IrMn layer can exist as exchange spring and thus might possibly mediate the long-range interlayer exchange coupling across the IrMn layer. However, no direct observation of a spiral AF spin structure has been made in the current study. The static magnetic measurements also reveal the long-range interlayer exchange coupling across the IrMn layer both at room temperature and low temperature for *t*_IrMn_ ≤ 6 nm, in agreement with the dynamic measurements.

## Conclusion

In summary, we probed the spin dynamics and magnetization reversal of the FM/AF/FM trilayer system CoFeB(10 nm)/IrMn(*t*_IrMn_)/CoFeB(10 nm). We report long-range dynamic exchange coupling between two CoFeB layers mediated by an IrMn spacer layer (up to *t*_IrMn_ = 6 nm). The study clearly shows the influence of the interlayer exchange coupling on *H_k_* and 4π*M*_eff_ values of the trilayers with *t*_IrMn_ as a control parameter. With the increase in *t*_IrMn_, the effective magnetic damping constant α_eff_ of the trilayers shows a rapid enhancement from 0.0074 to 0.0237 and is mainly associated with the combined influence of spin pumping into the IrMn spacer layer and weak interlayer exchange-coupling effects. In addition to intrinsic damping, we discussed the extrinsic contribution to spin relaxation originating from two-magnon scattering. In trilayers with *t*_IrMn_ = 2 nm, the linear dependence of α_eff_ on 1/*t*_CoFeB_ verifies that the damping is dominated by spin pumping and the resulting AF-induced interfacial damping parameter is found to be ca 0.08·10^−9^ m^−1^. The metastable AF domain state gives rise to asymmetric hysteresis loop and training effect at low temperature after field cooling. The study thus promises new opportunities for efficient spin transport in FM- and AF-based devices and allows effective tuning of the damping constant over a wide range.
